# Patients with Discordant Responses to Antiretroviral Therapy Have Impaired Killing of HIV-Infected T Cells

**DOI:** 10.1371/journal.ppat.1001213

**Published:** 2010-11-24

**Authors:** Sekar Natesampillai, Zilin Nie, Nathan W. Cummins, Dirk Jochmans, Gary D. Bren, Jonathan B. Angel, Andrew D. Badley

**Affiliations:** 1 Division of Infectious Diseases, Mayo Clinic, Rochester, Minnesota, United States of America; 2 University of Michigan, Ann Arbor, Michigan, United States of America; 3 Tibotec BVBA, Mechelen, Belgium; 4 Immunodeficiency Clinic, Ottawa Hospital, Ottawa, Ontario, Canada; NIH/NIAID, United States of America

## Abstract

In medicine, understanding the pathophysiologic basis of exceptional circumstances has led to an enhanced understanding of biology. We have studied the circumstance of HIV-infected patients in whom antiretroviral therapy results in immunologic benefit, despite virologic failure. In such patients, two protease mutations, I54V and V82A, occur more frequently. Expressing HIV protease containing these mutations resulted in less cell death, caspase activation, and nuclear fragmentation than wild type (WT) HIV protease or HIV protease containing other mutations. The impaired induction of cell death was also associated with impaired cleavage of procaspase 8, a requisite event for HIV protease mediated cell death. Primary CD4 T cells expressing I54V or V82A protease underwent less cell death than with WT or other mutant proteases. Human T cells infected with HIV containing these mutations underwent less cell death and less Casp8p41 production than WT or HIV containing other protease mutations, despite similar degrees of viral replication. The reductions in cell death occurred both within infected cells, as well as in uninfected bystander cells. These data indicate that single point mutations within HIV protease which are selected *in vivo* can significantly impact the ability of HIV to kill CD4 T cells, while not impacting viral replication. Therefore, HIV protease regulates both HIV replication as well as HIV induced T cell depletion, the hallmark of HIV pathogenesis.

## Author Summary

Although most patients infected with HIV who have persistent viral replication will experience a decline in CD4 T cell number, this is not always the case. In a small subset of patients in whom ART fails to suppress viral replication, CD4 T cell counts do not fall, for unknown reasons. We identified that these patients have an increased frequency of selected protease mutations, which we call discordance associated mutations (DAMs). While wild type protease rapidly induces cell death, protease containing DAMs have an impaired ability to induce cell death, due to a selective defect in cleavage of caspase 8. Furthermore, viruses containing DAMs replicate as efficiently as wild type, yet fail to induce infected cell death. These results demonstrate an unanticipated role of protease in determining the immunologic outcome of HIV infection *in vitro* and *in vivo*.

## Introduction

Prior to the development of effective treatments for HIV infection, the overwhelming majority of HIV-infected patients experienced an inexorable depletion of CD4 T cells, resulting first in immunodeficiency, followed by development of one or more AIDS defining illnesses and then death. The advent of effective therapies, and the knowledge of how best to use them, led to a reversal of this pathway for a majority of patients. Following initiation, the desired response to antiretroviral therapy is a reduction in the level of viral replication, reflected by reduced plasma viremia, and an increase in CD4 T cell number.

The study of exceptional circumstances has lead to some of the most valuable advances in our understanding of HIV pathogenesis. For example, studying a subset of long term non-progressors identified the CCR5Δ32 mutation as a factor in disease progression, which in turn has lead to the development of chemokine receptor antagonists as therapies for HIV infection. Another exceptional circumstance in HIV infection is the situation of immunologic response and virologic non response to antiretroviral treatment. Such discordance has been intensely studied and found to occur in 10–20% of cases of patients initiating therapy [1]–[3] and is more likely to occur in patients receiving a protease inhibitor [3]. Immunologically, discordant patients have reduced rates of apoptosis of CD4 T cells [4], [5] and higher TREC content [6], suggesting improved thymic function. Other quantitative improvements in immune function have also been observed relative to patients with both immunologic and virologic failure: improved lymphoproliferative responses to recall antigens and HIV antigens, improved cytokine production and improved IFNγ production [7]–[9]. Of importance, such responses can be maintained for as long as five years [10], and are associated with a reduced risk of disease progression [11] and death [12] compared to non-responders. Despite few mechanistic studies aimed at understanding such discordance, it is accepted that in the presence of reduced drug susceptibility, a portion of benefit achieved from therapy is due to persistent antiviral activity, as well as reduced replicative capacity [13]. Of course this contention requires that reduced replicative capacity translates into reduced CD4 T cell losses, although this has never been specifically demonstrated.

Expression of HIV-1 protease in cells is intrinsically cytotoxic and this property was exploited to screen and develop the protease inhibitor class, which has become an important treatment for HIV-1 infected patients. Indeed, HIV protease has degenerate substrate specificity and various host cellular proteins are targets for HIV-1 protease which are then cleaved following HIV infection including: actin, macroglobulin, myosin, spectrin, vimentin, desmin, DNA fragmentation factors, filamin, eukaryotic translation initiation factor-4γ, fibronectin, microtubule–associated proteins and other proteins [14]. Since HIV protease is active in the cytoplasmic compartment of HIV-infected cells, the cytotoxic effects are directly or indirectly related to cleavage of one or more of these cellular proteins [15]. One substrate of HIV protease is procaspase 8, and the absence of procaspase 8 renders cells resistant to the cytotoxic effects of HIV protease [16]. Specifically at a molecular level, HIV protease cleaves procaspase 8 between two phenylalanine residues, F355 and F356, generating a novel caspase 8 fragment of 41 kd that we call Casp8p41 [17]. Expression of Casp8p41 is independently cytotoxic and induces a similar death pathway as HIV protease: mitochondrial depolarization, release of cytochrome c, activation of caspases 9 and 3, and DNA fragmentation [18]. Importantly, generation of Casp8p41 occurs only following HIV infection or ectopic expression of HIV protease and not following death induced by other HIV proteins, death receptor signaling or mitochondrial toxins [17]. Consequently, detecting Casp8p41 in cells from HIV-infected patients [17] and the ability of Casp8p41 levels to predict CD4 T cell losses [19], altogether suggests that HIV protease-mediated killing occurs *in vivo*, and is of direct relevance to HIV pathogens. Further, lymph nodes from HIV-infected patients with detectable viral replication also express Casp8p41, which colocalizes with both infected and apoptotic cells [20]. Altogether, therefore, HIV protease production of Casp8p41 is one mechanism by which HIV-infected T cells can die, that may contribute to the overall CD4 T cell loss which occurs *in vivo*. In the current study, we evaluated whether protease mutations which occur in patients with discordant antiretroviral responses differ in their ability to generate Casp8p41 and to cause cell death, compared to patients with concordant antiretroviral responses.

## Results

### Resistance profiles of HIV protease differ between patients with discordant versus concordant responses to ART

**Figure 1:**
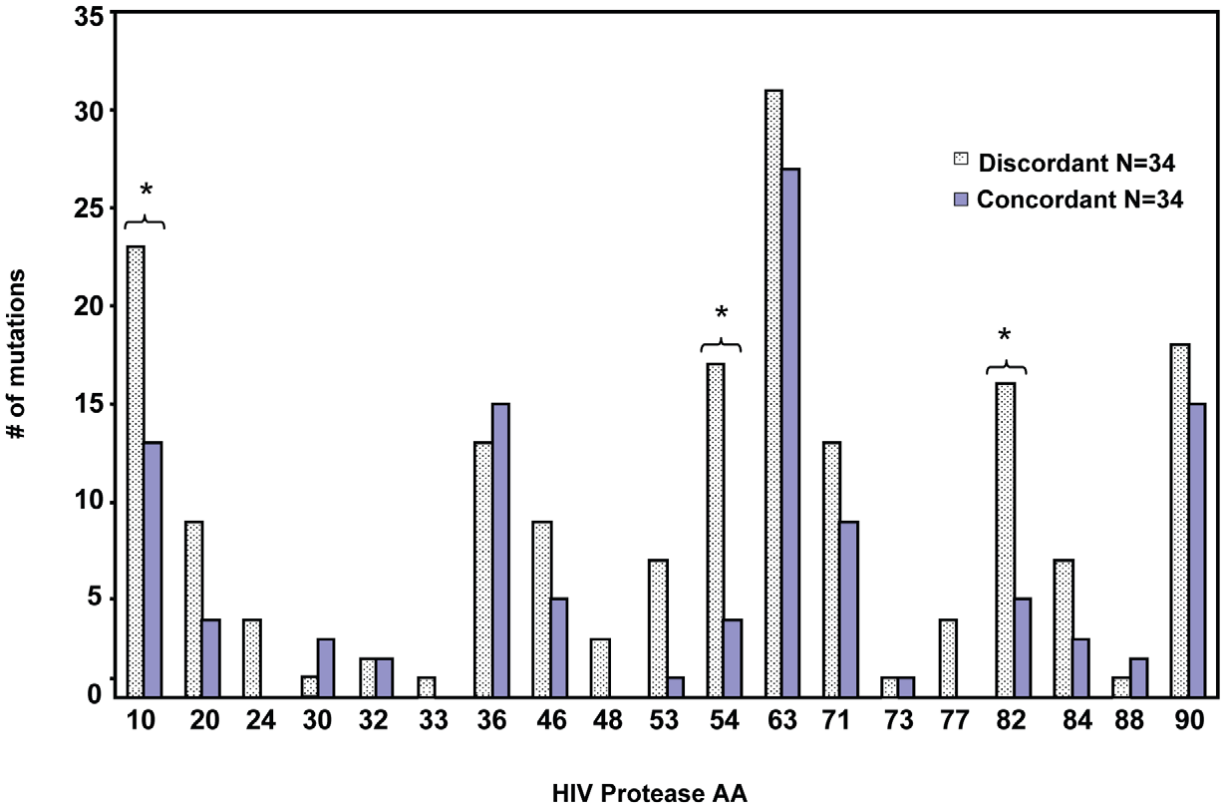
Protease resistance mutations differ between patients with concordant and discordant virologic failure. Protease genotype sequences were compared between 34 patients with concordant virologic failure, and 34 patients with discordant virologic failure, but immunologic improvement. *Indicates p value <0.05.

In response to subinhibitory concentrations of protease inhibitors, HIV protease resistance mutations are selected, which often involve both primary active site mutations that directly inhibit the action of PI, and a host of accessory resistance mutations that may occur far from the active site but may contribute to restoring the fitness or stability of protease activity [21]. We sought to determine whether the prevalence of PI mutations differed between patients with discordant virologic failure versus age matched ART treatment matched patients with concordant virologic failure. We used a strict definition of discordant failure [22], that is consistent with other published definitions [23] of increasing viral load over a period of three or more months, coincident with an increase in CD4 T cell number over the same timeframe in any patient receiving a combination of three or more antiviral agents. We compared the prevalence of mutations within protease of viral isolates from 34 patients with discordant virologic failure and immunologic responses, to 34 patients with virologic failure that was associated with immunologic decline. The clinical characteristics of these patients are described in Table 1. The median (interquartile range) increase in HIV viral load from the first measurement after initiating therapy to the peak after failure was1.17 (0.47–2.04) log_10_ copies/mL. Our rationale was that if protease mutations were unrelated to the generation of a discordant response, then the frequency of protease mutations should be similar between discordant patients and those with concordant failures. Conversely, if specific protease mutations predispose to the development of discordant response, then those mutations should occur with a significantly higher frequency in discordant compared to concordant virologic failures. Indeed in discordant patients a high frequency of mutations in protease were seen in selected sites that differed from that seen in patients with concordant failures. Differences were significant for mutations at amino acids 10 (p = 0.03) 54 (p = 0.001), and 82 (p = 0.008) (Figure 1). We termed these mutations Discordance Associated Mutations (DAMs).

**Table 1:** Demographics of discordant and concordant patients.

	Discordant (N = 34)	Concordant (N = 34)
Mean Age (range)	42.7 (24–58)	43.9 (26–55)
Sex (M∶F)	34∶0	32∶2
Change in CD4	+76 (15 to 286)	−113 (−22 to −325)
Change in VL (log)	+1.17 (0.47−2.04)	+1.03 (0.55–1.87)
Duration of detectable viremia (months)	9.2 (3 to 17)	8.1 (3 to 21)
# receiving a PI	34/34	34/34
Years diagnosed with HIV	6.1 (2–18)	5.2 (3–15)

### Selected protease mutations impair protease mediated cell killing

**Figure 2:**
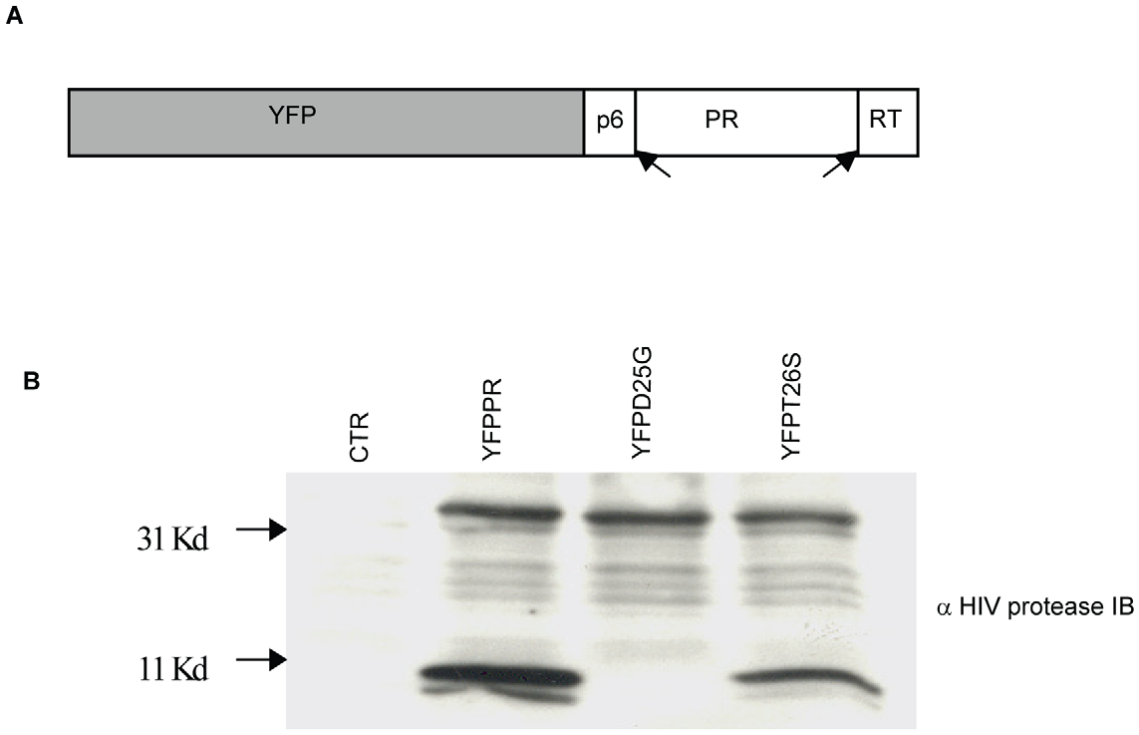
Subcloning HIV-1PR. HIV protease along with flanking regions within RT and P6 fused with YFP was cloned into a mammalian expression vector (A). Following expression, protease autocatalyses and cleaves itself out of the fusion protein creating an 11 Kd protease protein. This activity is absent in the D25G protease inactive construct, and is reduced in the T26S catalytically impaired construct (B).

We next tested whether DAMs reduce the ability of HIV protease to kill host cells relative to non-DAM mutations. For this we chose the DAMs I54V, V82A, and the non-DAMs K20R, L63P, D30N and L90M. A YFP-PR fusion construct was developed containing the p6 and RT cleavage sites which normally flank protease. Following expression, this YFP fusion construct auto-processes to generate mature 11 kd protease (Figure 2). Within this backbone, we introduced the point mutations by site directed mutagenesis. In addition, we introduced the control mutations of active site dead D25G, and T26S which has been shown to have reduced protease catalytic and cytotoxic activity [24].

**Figure 3:**
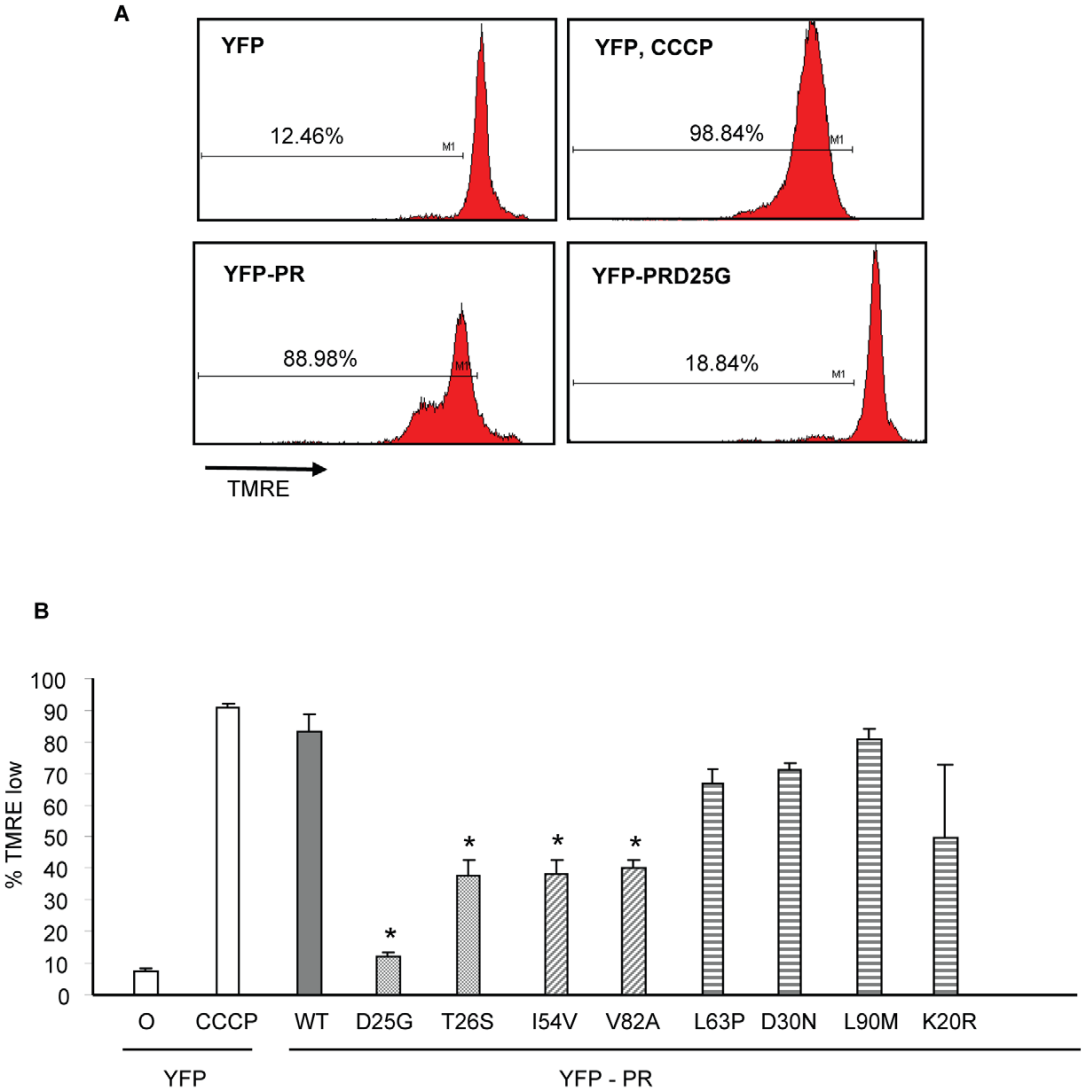
Discordance Associated Mutations (DAMs) within HIV-1PR alters the loss of mitochondrial transmembrane potential. YFP HIV protease or the indicated protease point mutants were expressed in HeLa cells, and the YFP positive cells were analyzed for loss of mitochondrial transmembrane potential (A, B). *Indicates p value ≤0.05. Data reflective of at least three independent replicates. Bars with solid color represent controls, bars with angled hatching represent DAMs, and bars with horizontal hatching represent non-DAMs.

We have previously shown that expression of HIV-1 PR causes cell death which is associated with loss of mitochondrial transmembrane potential, caspase 3 activation and nuclear fragmentation [16]. After transfection with YFP and YFP protease, He-La cells were assessed for loss of mitochondrial transmembrane potential in the YFP-PR positive cells by TMRE staining, which is a hydrophobic dye, taken up by mitochondria with intact transmembrane potential. We specifically evaluated TMRE loss in the YFP positive cells, indicating expression of protease. Expression of YFP alone or YFP with catalytically inactive YFP D25G PR resulted in minimal loss of transmembrane potential. Conversely, YFP-WT PR or treatment with CCCP, which uncouples mitochondrial transmembrane potential, resulted in significant loss of TMRE retention (Figure 3A). Strikingly, expression of YFP-L63P PR, YFP-D30N PR, YFP-K20R-PR, or YFP-L90M PR caused high degrees of transmembrane potential loss, similar to WT Protease. In contrast, YFP-PR fusions containing the DAMs I54V, V82A caused a lesser degree of transmembrane potential loss than WT-PR, that was similar to that of catalytically impaired T26S (Figure 3B).

**Figure 4:**
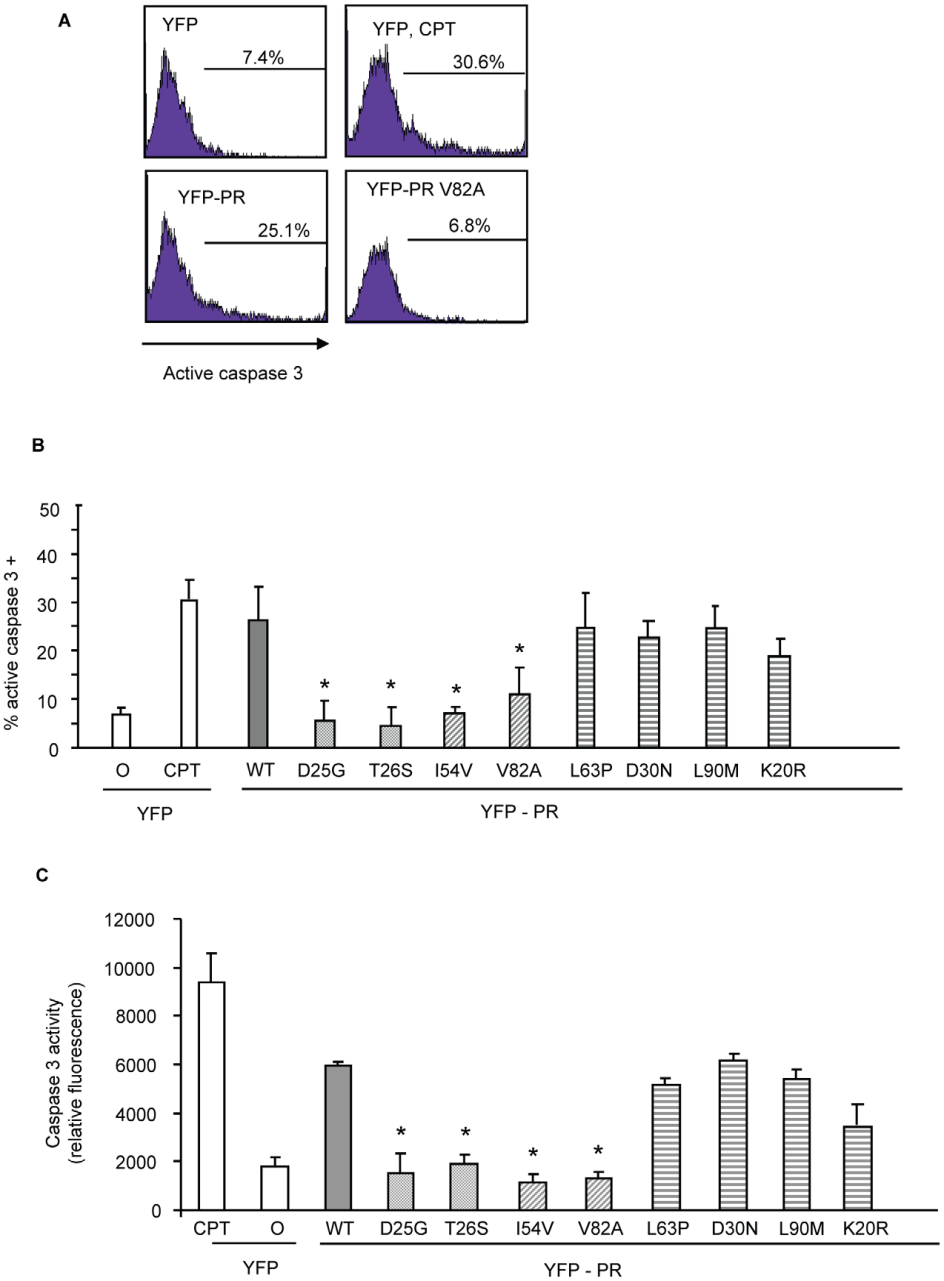
Discordance Associated Mutations (DAMs) within HIV-1PR alters the generation of caspase 3. YFP HIV protease or the indicated protease point mutants were expressed in HeLa cells, and the YFP positive cells were analyzed for percent active caspase 3 positive (A, B), and total caspase 3 activity (C). *Indicates p value ≤0.05. Data reflective of at least three independent replicates. Bars with solid color represent controls, bars with angled hatching represent DAMs, and bars with horizontal hatching represent non-DAMs.

**Figure 5:**
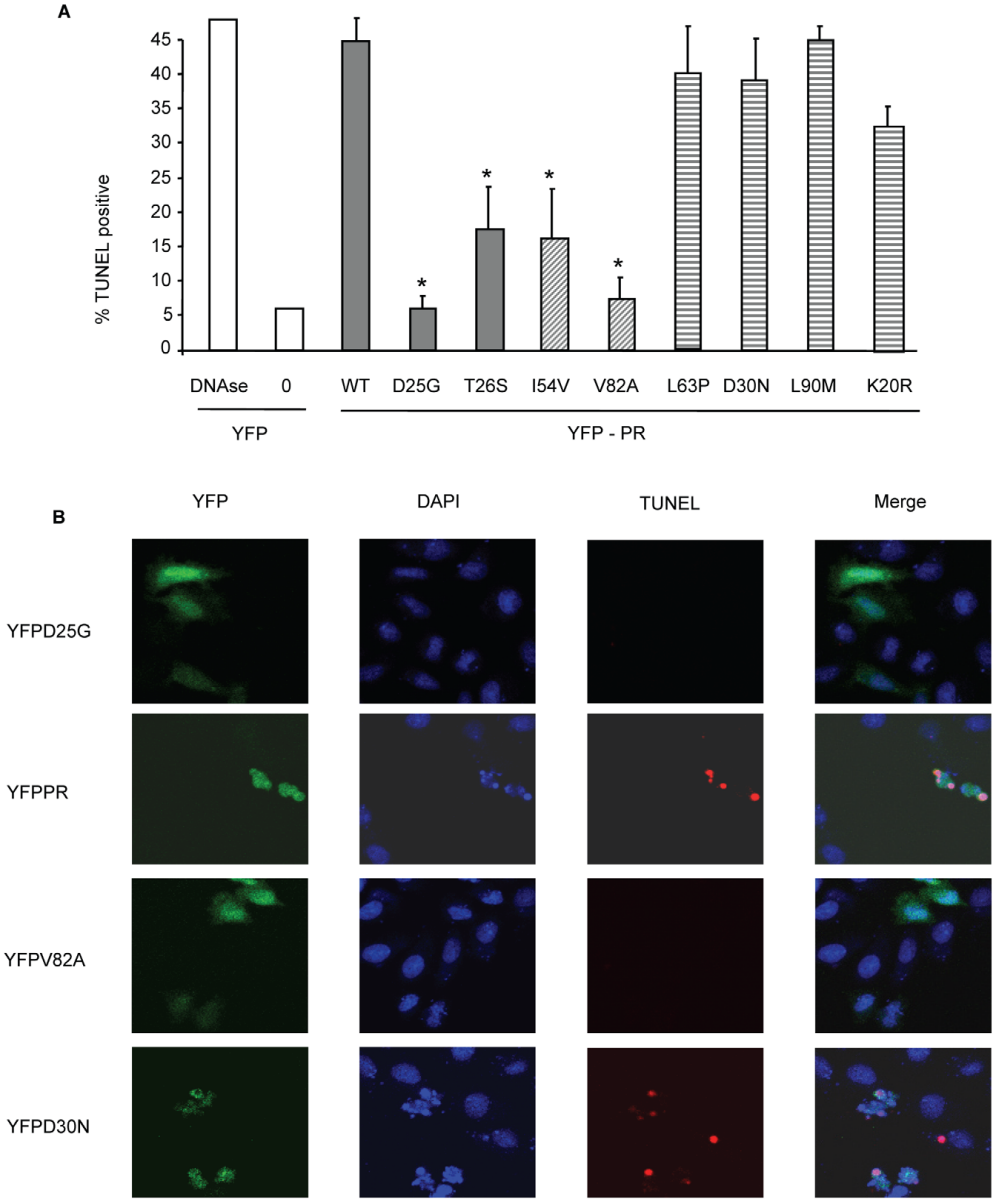
Discordance Associated Mutations (DAMs) within HIV-1PR alters the generation of TUNEL positivity. YFP HIV protease or the indicated protease point mutants were expressed in HeLa cells, and the YFP positive cells were analyzed for TUNEL positivity (A, B). *Indicates p value ≤0.05. Data reflective of at least three independent replicates. Bars with solid color represent controls, bars with angled hatching represent DAMs, and bars with horizontal hatching represent non-DAMs.

The putative impairment in cytotoxic activity of I54V and V82A were next assessed by an independent measure of death, generation of active caspase 3, and consequent caspase 3 activity. Consistent with previous findings, WT protease expression generated a significant amount of active caspase 3, and of caspase 3 activity (Figure 4A, 4B, 4C). Mirroring the results of TMRE staining, I54V and V82A had reduced generation of active caspase 3 and caspase 3 activity than WT protease, while K20R, L63P, D30N, L90M, generated similar levels of active caspase 3 and of caspase 3 activity as WT (Figure 4A, 4B, 4C). TUNEL staining confirmed these observations (Figure 5A, 5B) seen with TMRE staining and caspase 3 activity assays; the DAMs I54V, and V82A are associated with reduced induction of cell death compared to WT or the non-DAMs L63P, D30N, L90M, or K20R.

**Figure 6:**
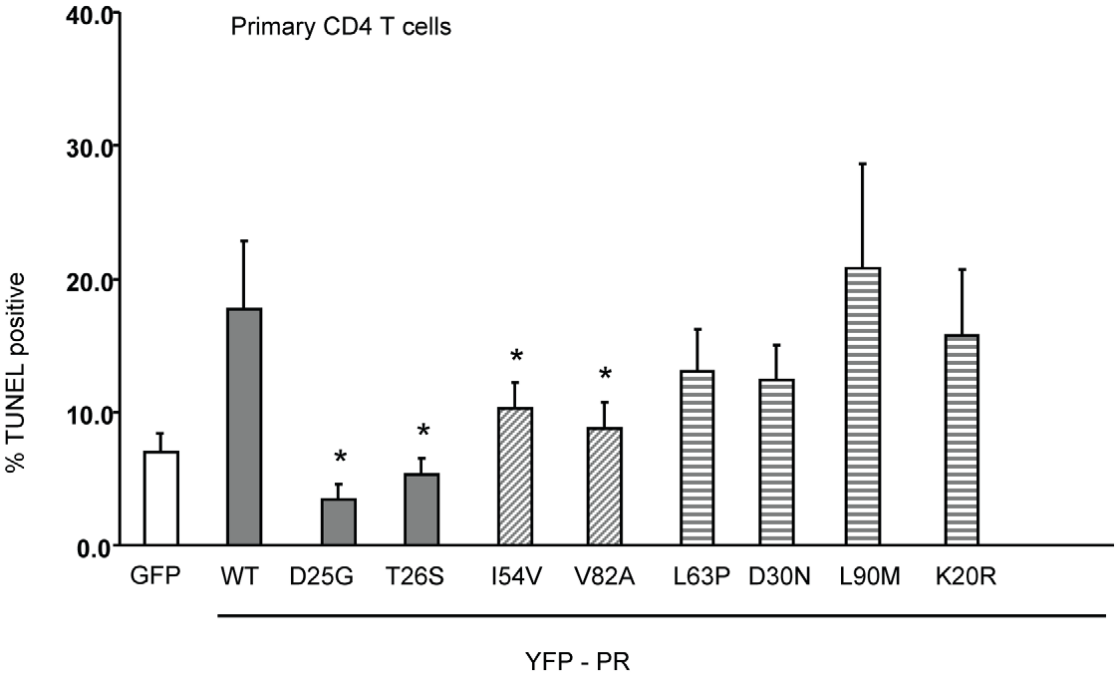
Expression of Discordance Associated Mutations (DAMs) in primary CD4 T cells alters generation of TUNEL positivity. The same constructs were used to transfect primary CD4 T cells, and the YFP positive cells analyzed for TUNEL positivity. *Indicates p value ≤0.05. Data reflective of at least three independent replicates. Bars with solid color represent controls, bars with angled hatching represent DAMs, and bars with horizontal hatching represent non-DAMs.

In order to examine the relevance of these differences to primary CD4 T cell death, we next transfected primary CD4 T cells with the same constructs and analyzed death by TUNEL positivity. YFP positive primary CD4 T cells containing I54V or V82A protease had significantly fewer apoptotic cells than YFP positive primary CD4 T cells containing WT, K20R, L63P, D30N or L90M protease (Figure 6). Altogether, these data confirm the underlying observation that the DAMs I54V and V82A cause less cell death than either WT HIV PR, or the other non-DAM mutants tested, confirming the reduced ability of protease containing DAMs to kill infected cells.

### HIV PR mutations that impair cell killing generate less Casp8p41 in primary CD4 T cells

**Figure 7:**
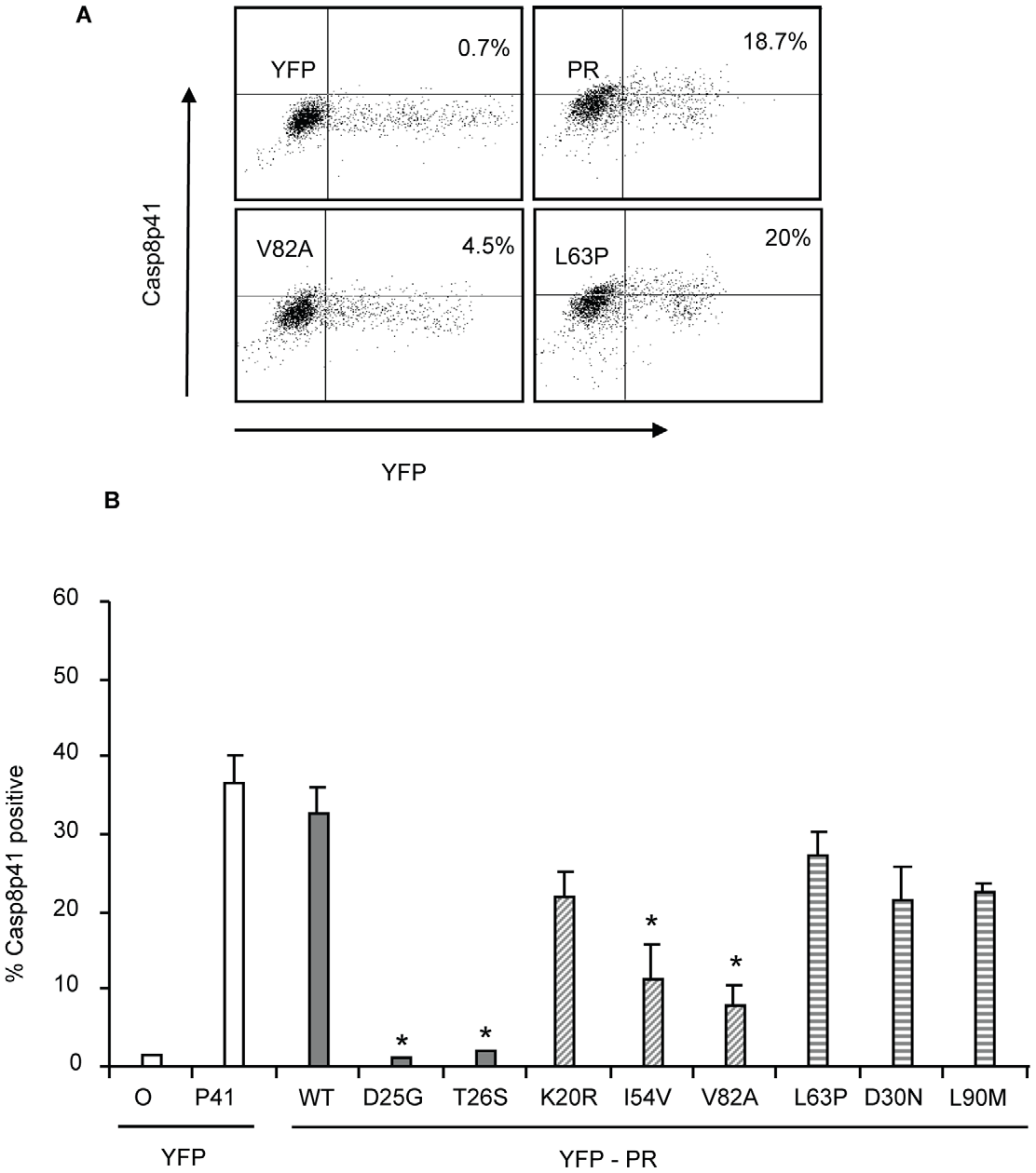
Reduced HIV-1PR-induced cell death is associated with reduced Casp8p41 production. YFP HIV protease or the indicated protease mutants were expressed in primary CD4 T cells and YFP positive cells were analyzed for Casp8p41 positivity (A, B). *Indicates p value ≤0.05. Data reflective of at least three independent replicates. Bars with solid color represent controls, bars with angled hatching represent Discordance Associated Mutations (DAMs), and bars with horizontal hatching represent non-DAMs.

Our previous publications indicate that HIV PR mediated killing requires procaspase 8 cleavage with subsequent generation of Casp8p41 [16]–[18]. Therefore, if our model holds, the impaired cell killing associated with I54V and V82A protease expression, should be associated with less Casp8p41 production. To test this hypothesis, we expressed mutant or WT HIV protease in primary CD4 T cells, and analyzed Casp8p41 production using a neoepitope specific Casp8p41 antibody [17]. Whereas expression of YFP WT protease in primary CD4 T cells resulted in a significant proportion of Casp8p41 positive cells, expression of either I54V or V82A resulted in significantly less Casp8p41 positivity than K20R, L63P, D30N, or L90M (Figure 7A, 7B).

### *In vitro* cleavage of procaspase 8 vs. gag-pol sequences by wild type or mutant PR

We next assessed the ability of HIV protease to cleave procaspase 8 to produce Casp8p41. We also compared the ability of these protease constructs to cleave procaspase 8 relative to their ability to cleave gag-pol, in order to understand whether the reduced Casp8p41 production was due to a selective inability of protease to cleave that substrate, or a global reduction in catalytic activity. Two different 12 amino acid peptides were constructed reflecting the 12 amino acids surrounding the protease cleavage sites within caspase 8 and gag-pol. These peptides were generated containing fluorescence resonance energy transfer peptides; a DABCYL fluorescence acceptor group at the N terminus, and a C terminal EDANS fluorescence donor group. In this system, the DABCYL group acts to quench the EDANS fluorophore. Upon cleavage into two separate fragments by HIV-1 protease (at the Phe-Phe in Casp8p41 or at the Tyr-Pro in gag-pol), the fluorescence of EDANS is unrepressed and peptide cleavage is monitored by increasing fluorescence emission.

**Figure 8:**
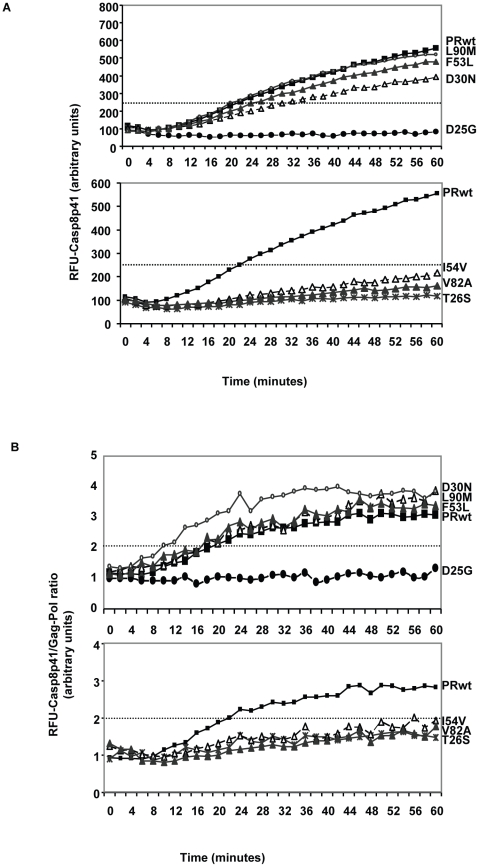
Recombinant HIV protease containing Discordance Associated Mutations (DAMs) has an impaired ability to cleave procaspase8 relative to gag-pol. HIV protease was produced in *e.coli*, and wild type protease, or the indicated point mutants were incubated with a fluorogenic peptide corresponding to the site in procaspase8 which is cleaved by HIV protease, and cleavage monitored over time by measuring fluorescence release (A). In order to normalize for the amount and activity of the protease preparations, HIV protease used above was also analyzed for gag-pol cleavage by using a fluorogenic peptide corresponding to the gag-pol cleavage site. At each time point a ratio was calculated by dividing the fluorescence released in the procaspase 8 cleavage assay with the amount of fluorescence released in the gag-pol cleavage assay. The ratio is depicted in (B).

We tested WT HIV protease or the following point mutations: D25G (active site dead), T26S (catalytically impaired), D30N, F53L, or L90M; or the DAMs I54V and V82A produced in E-coli. Each protease preparation was reacted against either the Casp8p41 substrate or the gag-pol substrate. Of interest, the two mutations (I54V and V82A) that are over represented in the discordant subjects had reduced cleavage of the Casp8p41 substrate compared to WT protease or D30N, F53L, or L90M, suggesting that these mutations have a selective impairment in the ability to generate Casp8p41, and therefore, an impairment in the ability to induce Casp8p41-dependent death (Figure 8A). In order to control for the amount of protease tested in these assays, a separate series of experiments were performed. Equal amounts of recombinant protease were used to cleave the Casp8p41 substrate as the gag-pol substrate. These results were used to calculate a ratio of Casp8p41 cleavage relative to gag-pol cleavage. Strikingly, only the I54V and V82A DAM mutations produced a ratio of Casp8p41:gag-pol cleavage that was significantly less than the ratio that was produced by WT HIV protease. By contrast, D30N, F53L, and L90M all produced Casp8p41:gag-pol cleavage ratios greater than WT protease (Figure 8B).

### Impact of DAMs on outcome of HIV infection

**Figure 9:**
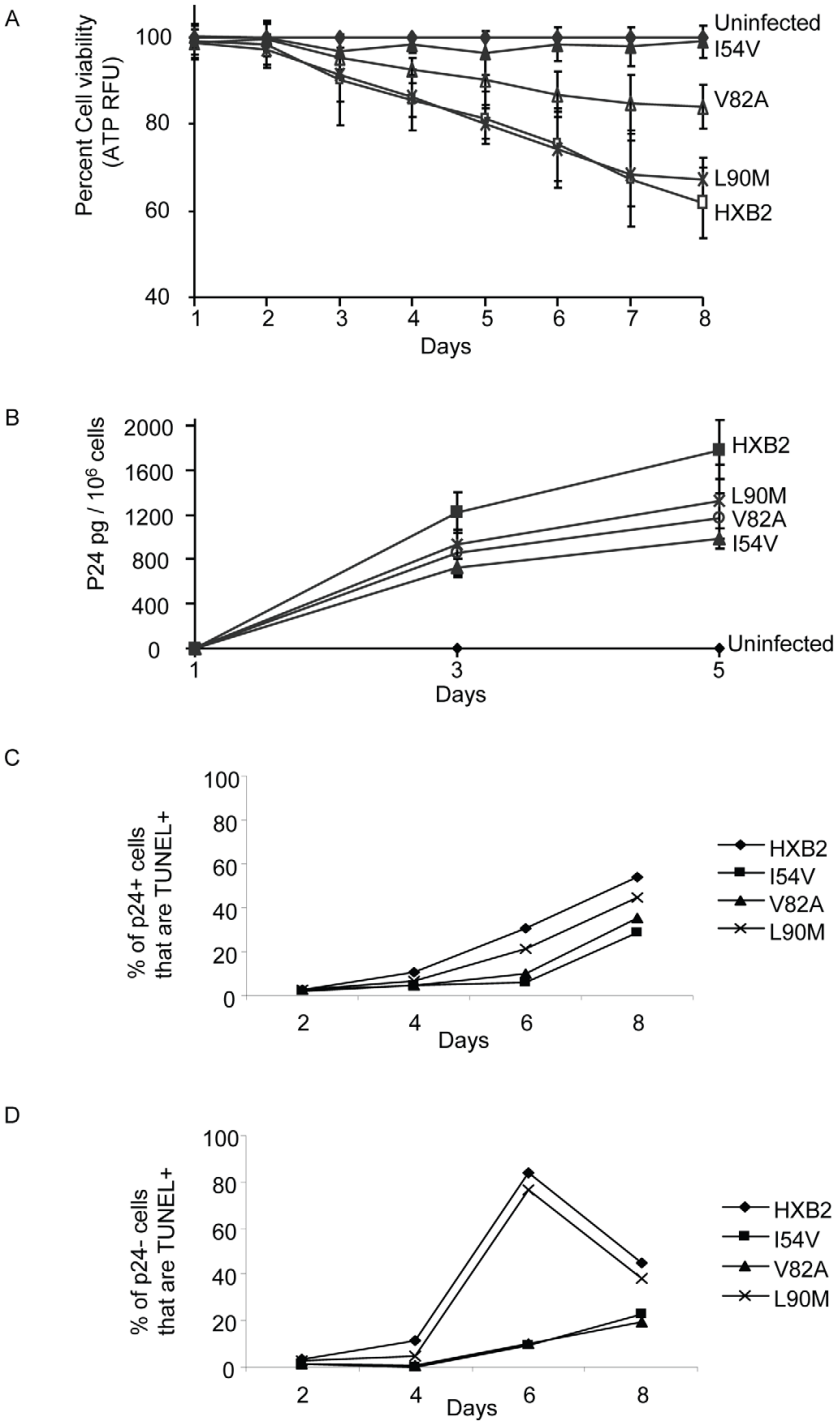
HXB2 HIV containing Discordance Associated Mutations (DAMs) are associated with impaired killing of primary CD4 T cells. Primary CD4 T cells were infected with HXB2 containing wild-type HIV protease, or protease containing the mutations L90M, I54V, or V82A, and analyzed for (A) cell viability by ATP production, (B) P24 production, and (C) TUNEL positivity in the infected (P24 +), or (D) uninfected (P24-) subsets.

We have presented evidence that the DAM mutations I54V and V82A, have an impaired ability to cleave procaspase 8 relative to gag-pol. This would predict that viruses containing the mutations should have preserved viral replication, yet a selective impairment in Casp8p41 production and reduced killing of HIV-infected cells. Therefore, we performed HXB2 virus infections of the human T cell lymphoblastoid line MT4, and measured cell viability, Casp8p41 content, and apoptosis by TUNEL positivity in the HIV-infected cells, as well as viral replication kinetics. Infection of MT4 cells with WT HXB2 virus resulted in a time-dependent decline in total cell viability, such that by Day 8 post-infection total cell viability approaches 60%, whereas uninfected control cell viability remained >95% (Figure 9A). Infection with HXB2 containing the non-discordance associated mutation L90M caused a similar degree of loss of total cell viability, whereas infections with HXB2 containing the discordance associated mutations I54V or V82A caused significantly less loss of total cell viability. These differences in total cell viability occurred despite similar levels of viral replication as reflected by similar levels of P24 antigen present in the culture supernatants, from the infections with the L90M, V82A, or I54V virus infections (Figure 9B).

**Figure 10:**
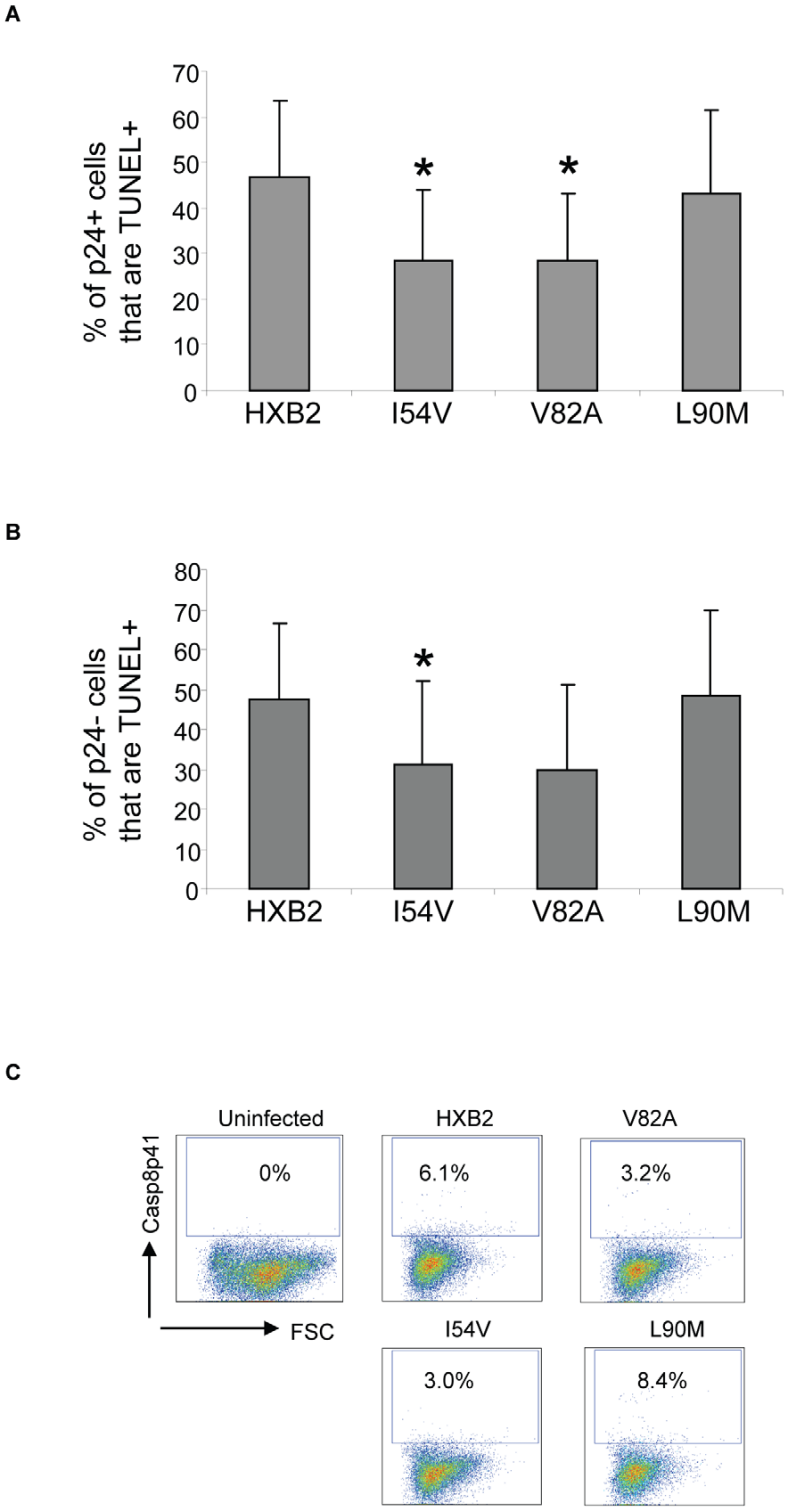
HXB2 containing Discordance Associated Mutations (DAMs) are associated with reduced generation of TUNEL positivity and reduced Casp8p41 production. Cells were infected as in Figure 9 and analyzed for TUNEL positivity in the P24 positive (A) or P21 negative (B) cells, as well as for Casp8p41 (C). Results in (A) and (B) are pooled results from three independent experiments. Data in (C) is reflective of three independent replicates.

We next specifically analyzed cell death induced in HIV-infected cells, as well as in uninfected bystander cells by co-staining with TUNEL and P24 antigen, and comparing death in the infected (p24 positive, Figure 9C) and uninfected (p24 negative, Figure 9D) T cell subsets. Consistent with the premise that mutations in HIV protease will reduce the ability of HIV protease to kill infected cells, protease containing V82A or I54V induced less apoptosis in infected cells than either WT or L90M (Figure 10A). Of interest this trend was also observed in the uninfected cells, with less uninfected cell killing being observed with I54V, and a trend towards less death in the V82A protease mutant (Figure 10B).

The measurements of total cell viability reflect cell death induced by all proapoptotic factors present in HIV-infected cell cultures, including Tat, Nef, Env, Fas ligand, TRAIL, Casp8p41, and others. As our underlying premise is that alterations in protease will impact Casp8p41 induced death, which we have previously shown to occur only within the infected cell subset, we specifically analyzed Casp8p41 content (Figure 10C). Casp8p41 content was greatest in HXB2 WT infected cells and in the L90M non-DAM infected cells. Consistent with our *in vitro* data infection with HIV containing the DAM mutations I54V or V82A resulted in reduced generation of Casp8p41.

## Discussion

In the current study we have identified mutations within HIV protease which occur with an increased frequency in patients who manifest a discordant response to antiretroviral therapy, and have evaluated whether those mutations within HIV PR alter the generation of Casp8p41 and consequently the rate of Casp8p41 induced death. Those studies show that the mutations I54V and V82A cause less procaspase 8 cleavage, less Casp8p41 production, and impaired induction of cell death following isolated protease expression or HIV infection. This manifests as a reduced proportion of cell death in cells which are directly infected with HIV as well as a reduced proportion of cell death in the bystander (uninfected cell) population. Reasons for less cell death in the uninfected cells are unclear, but might involve an altered cytokine milieu which is associated with reduced priming of cells towards an apoptosis susceptible phenotype. A possible pathway by which this might occur is through HIV infected cells releasing paracrine factors when they die, which promotes an activated or apoptosis susceptible population of bystander cells. In such a scenario, when infected cell death is reduced, bystander death would likely also be reduced. Soluble factors which might contribute to such an effort on bystander cells include mitochondrial DNA, which is recognized by innate mechanisms as a damage associated molecular pattern, which leads to activation, inflammation and AICD [25]. This might explain why others have reported changes in CD8 T cell activation in patients who develop PI mutations with out significant changes in viral replication [26].

It has previously been suggested that Protease mutations might impact the ability of HIV to kill CD4 T cells, through unknown mechanisms [27], [28]. Finding that certain isolated mutations within protease result in an impaired ability of protease to cleave procaspase 8, and induce cell death, coupled with knowledge that multiple mutations within protease cooperatively impact viral replicative fitness [29], [30], suggests that multiple mutations within protease will likely influence the ability of protease to induce cell death in a cooperative manner. Indeed the combined effect of mutations which impair protease cytotoxic effects (e.g., I54V and V82A) along with other mutations that do not (e.g., L63P, L90M) will need to be individually assessed. In this light it is of great interest that I54V and V82A have evidence of having co-evolved; possibly by virtue of cooperative selection due to low Casp8p41 production [31]. Similarly the effects of mutations not residing within protease itself, but influencing protease processing (e.g., gag-pol cleavage site mutations) will require testing.

The mutation L10I is also of interest, in that it caused an intermediate degree of procaspase 8 cleavage generating less Casp8p41 than WT protease, but more than I54V and V82A (data not shown). Furthermore, expression of L10I caused less protease induced death than WT protease, more than I54V and V82A, albeit neither difference reached significance. This observation suggests that there are likely degrees of impairment in cell death afforded by different mutations.

It is generally agreed that the proportion of infected CD4 T cells [32], [33] is insufficient to explain the magnitude of CD4 T cell loss that is seen during HIV infection. Although this conclusion is almost certainly correct, further lines of evidence suggest that the proportion of infected CD4 T cells that die might be higher than originally suggested. This belief stems from several lines of reasoning. First, presuming the situation where the majority of infected cells die, then nucleic acid-based techniques to identify infected cells would predictably lead to underestimates. This is due to recent knowledge that during the terminal stages of apoptosis, DNA and RNA is fragmented and consequently TUNEL assays become positive. During this period of time when DNA and RNA are cleaved, assays which rely upon detection of large (>2 kb) HIV specific sequences as means of identifying infected cells would predictably cause false negative results. Second, early assays used to identify HIV-infected cells based upon intracellular staining with anti-p24 antibodies consistently identified a significant proportion of CD4 T cells as containing p24; in many cases 1 to 3% of CD4 T cells contained p24 [34]–[36]. Third, studies using GFP reporter cell lines or GFP labeled virus [37], [38] infected primary resting CD4 T cells consistently indicate greater than 20% of target cells are infected by HIV *in vitro*; it seems very unlikely, therefore, that well less than 0.1% of susceptible target cells would be infected *in vivo*. Fourth, we have analyzed Casp8p41 content in cells from HIV-infected patients both within the peripheral blood and lymphoid tissues, and have consistently demonstrated as many at 1–3% of CD4 T cells in the peripheral blood of HIV-infected patients containing HIV-specific marker Casp8p41 [17]. Therefore, in our view, it is likely that a proportion of CD4 T cells are infected *in vivo* and that some of these die through Casp8p41-dependent mechanisms. The *in vivo* role of infected cell killing is further supported by recent data whereby HIV infects and kills CD4 T cells *in vivo* [39]. However, it still remains unknown what the relative contribution of infected cell killing to uninfected cell killing is *in vivo*. Data contained in the current report suggests however that patients harboring selected protease mutations might have a higher proportion of infected cells that do not die through this Casp8p41 mechanism.

Identification of Casp8p41 as a mechanism by which infected, but not uninfected, cells die following HIV infection, along with the current observation that HIV PR resistance is associated with impaired CD4 T cell killing, allows a potential explanation for observations that arose during a period when PI drug resistance was common. Physicians who treated HIV-infected patients during that era consistently observed impaired CD4 T cell losses, despite ongoing viral replication [40]–[42]. Demonstrating that select protease inhibitor resistant mutations are associated with impaired CD4 T cell killing and impaired Casp8p41 allows the possibility that changes in Casp8p41 production might impact CD4 T cell depletion. Future studies aimed at evaluating the cooperative effects of multiple mutations within protease on Casp8p41 production, along with longitudinal evaluations of Casp8p41 as a predictor of CD4 T cell change should help answer this question.

## Materials and Methods

### Patient samples

Patient genotypes were abstracted from the clinical records of patients attending the immunodeficiency clinic of the University of Ottawa. Patients were selected in 2002 based upon a rising viral load over a period of three or more months while receiving three or more antiretroviral agents. When CD4 counts rose over that period, they were classified as discordant virologic failures, as we have previously described [22]. When CD4 counts fell over that time period they were classified as concordant virologic failures. This protocol was reviewed and approved by the IRB of the University of Ottawa. Written informed consent was waived as samples were de-identified. All patients provided oral consent, as required by the IRB, which was documented.

### HXB2 HIV containing protease mutations

HIV (HXB2) viral stocks containing either the WT protease gene or mutations coding for single amino acid substitutions (I54V, V82A, or L90M) of the protease gene were produced as follows: Mutant PR coding sequences were generated from a pGEM vector containing the HIV-1 LAI (clone HXB2) PR and RT coding sequence, using the QuikChange Site-Directed Mutagenesis Kit (Stratagene, Foster City, CA) and high-performance liquid chromatography-purified primers (Genset Oligos). Genotyping of the HIV coding sequences in the plasmids upon site-directed mutagenesis confirmed the presence of the desired change(s). HXB2 proviral clone [43]. The presence of the desired mutation(s) in the recombinant viruses was confirmed by genotyping. The HIV-1 viral stocks were propagated by infecting MT4 cells. After 24 hours, infected MT4 cells were washed and resuspended with complete RPMI. The cells were incubated and when cytopathic effects occurred, the recombinant viruses were harvested and viral concentration measured by p24 ELISA. HIV protease sequences were verified by RT-PCR followed by DNA sequencing. Equal amounts of virus as determined by p24 were stored at −80°C or used directly to infect primary CD4 cells.

### Cell culture, transfection and Western blot

Primary CD4 T cells were harvested from peripheral blood, and separated by negative selection using RosetteSep (Stem cell technologies). Transfection of primary human Cd4 T cells was accomplished using Amaxa Nucleofector. HeLa cells were purchased from ATCC (Manassas, VA). Cells were maintained in Dulbecco Modified Eagle Medium (DMEM) or RPMI1640 (GIBCO, Carlsbad, CA) supplemented with 10% (v/v) FBS, 1% penicillin and streptomycin, and 2 mM L-glutamine, and incubated at 37°C in a humidified atmosphere containing 5% CO_2_. To transfect HeLa cells, exponentially growing cells were transfected with DNA plasmids isolated from CsCl gradients and delivered into cells with Fugene 6 at a ratio of 1 µg DNA: 2 µl Fugene 6. For Western blot analysis, 50 µg of cytosolic proteins were fractionated on a 15% polyacrylamide gel, then transferred onto PVDF membrane (Millipore, Bedford, MA) for 3 hours at 1000 mA using transfer buffer (25 mM Tris, 192 mM glycine). The membranes were blocked by incubation in TBS buffer (20 mM Tris, 150 mM NaCl, 0.05% Tween, pH 7.5) containing 2% BSA overnight at 4°C and washed five times with TBS buffer. Then the membranes were blotted for 1 hour at room temperature with a primary antibody against HIV protease (AIDS Reagent Program). The blots were washed five times with TBS and developed with an HRP linked secondary antibody. The blot was developed using SuperSignal (Pierce, Rockford, IL) following the manufacturer's protocol.

### Plasmid construction and DNA sequencing

YFP-HIV-1 PR was constructed by inserting a PCR product developed from the WT HxB2 provirus plasmid encompassing the PR gene plus 69 base pairs upstream and 60 base pairs downstream of the PR gene into the EcoRI/BamHI sites of pEYFP-C1 (Clontech, Mountain View, CA). The PR sequence was confirmed within the EYFP open reading frame by DNA sequencing at Mayo Clinic's Molecular Core Facility. The PR mutants, L10I, K20R, D25G, T26S, D30N, I54V, L63P, V82A and L90M were created on the pEYFP-C1PR plasmid with the Quick-Exchange Site-directed Mutagenesis Kit (Stratagene, La Jolla, CA). The primers used are as follows:

K20R sense: 5′-GGGCAACTAAGGGAAGCTCTA-3′, anti-sense: 5′-TAGAGCTTCCCTTAGTTGCCC-3′; D25G sense: 5′-AGGAAGCTCTATTAGGTACAGGAGCAGATGA-3′, anti-sense: 5′-TCATCTGCTCCTGTACCTAATAGAGCTTCCT; T26S sense: 5′-GAAGCTCTATTAGATAGTGGAGCAGATGATACA-3′; anti-sense: 5′-TGTATCATCTGCTCCACTATCTAATAGAGCTTC-3′; I54V sense: 5′-GGAATTGGAGGTCTTATCAAAGTAAGA-3; anti-sense: 5′-TCTTACTTTGATAAGACCTCCAATTCC-3′; I54V sense: 5′-ATTGGAGGTTTTGTCAAAGTAAGACAG-3′, anti-sense: 5′-CTGTCTTACTTTGACAAAACCTCCAAT-3′; L63P sense: 5′-GTAAGACAGTATGATCAGATACCCATAGAAATCTGTGGAC-3′; anti-sense: 5′-GTCCACAGATTTCTATGGGTATCTGATCATACTGTCTTAC-3′; V82A sense: 5′-GTAGGACCTACACCTGCCAACATAATTGGAAG-3′; anti-sense: CTTCCAATTATGTTGGCAGGTGTAGGTCCTAC-3′; L90M sense: 5′-TAATTGGAAGAAATCTGATGACTCAGATTGGTTG-3′;and anti-sense: 5′-CAACCAATCTGAGTCATCAGATTTCTTCCAATTA-3′. All primer synthesis and mutation confirmation was carried out in Mayo Clinic's Molecular Core Facility.

### Measurement of mitochondrial membrane potential (MMP), TUNEL and Casp8p41 by flow cytometry

Mitochondrial membrane potential (MMP) of the YFP and YFP fusion transfected cells was measured with the mitochondrial specific red fluorescent dye, tetramethylrhodamine, ethyl ester, perchlorate (TMRE) [44]. Briefly, 100 nM TMRE was added to 1×10^6^ cells in 1 ml PBS at 37°C for 20 minutes. TMRE and YFP double positive cells were analyzed by flow cytometry at 20,000 events per sample. To measure apoptosis, the *In Situ* Cell Death Detection kit (Roche Applied Science, Indianapolis, IN) was used to stain 3′ DNA strand breaks following the manufacturer's protocol. Briefly, CD4 T cells were fixed in 2% paraformaldehyde, and permeabilized in 0.1% sodium citrate containing 0.1% Triton X-100 for 5 minutes. Fixed cells were resuspended in 50 µl of TUNEL reaction buffer containing PE-dUTP and 5 U TdT, and analyzed by flow cytometry. To determine the expression of Casp8p41, 10∧6 peripheral blood lymphocytes were fixed with 2% paraformaldehyde in PBS then permeabilized with PBS plus 0.1% NP-40 on ice for 2 minutes and then incubated with Alexa 647- or PE-labeled Casp8p41 antibody [17] for 1 hour. Flow cytometry was performed using a FACScan (Becton Dickinson, Franklin Lakes, NJ) and analyzed using CellQuest software.

### Confocal microscopy

HeLa cells were seeded onto glass coverslips and transfected with plasmids coding for YFP-HIV protease WT or mutants. TUNEL staining for detection of apoptosis was done according to the manufacturer's protocol (Roche, Nutley, NJ). DAPI was added to identify nuclei. Laser-scanning confocal microscopy was performed using a Zeiss LSM-510 (Carl Zeiss Inc., Thornwood, NY).

### Caspase 3/7 activity detection

Caspase 3/7 activity in HIV-1 PR WT and mutant transfected HeLa cells was measured with a Caspase 3 Fluorometric Assay Kit (R&D Systems, Inc., Minneapolis, MN) following the manufacturer's instructions. Briefly, the cells were harvested at 6–8 hours after transfection, lysed, and equal amounts of protein were added to each reaction in a 96 flat well microplate. The fluorescent signals were read on a Microplate Fluorescent Reader (FL600, Bio-Tek, Winooski, VT) using excitation and emission wavelengths of 340 and 450 nm, respectively.

### HIV-1 protease activity assay

To assess the effect of the various mutations on the proteolytic activity of HIV protease, a fluorometric assay was devised to compare the relative rates of cleavage of a consensus gag-pol site with the caspase 8 site. The fluorogenic peptides (gag-pol: Arg-Glu(EDANS)-Ser-Gln-Asn-Tyr-Pro-Ile-Val-Gln-Lys(DABCYL)-Arg; caspase 8: Arg-Glu(EDANS)-Pro-Lys-Val-Phe-Phe-Ile-Gln-Ala-Lys(DABCYL)-Arg) were synthesized in the Mayo Protein Core Facility such that fluorescence would be quenched until the internal peptide is cleaved. Measurements were performed in 96-well plates with a SPECTRAmax GeminiXPS spectrofluorometer (Molecular Devices, Sunnyvale, CA). For each enzymatic reaction, 50 µl of cell lysates containing HIV protease was added to 150 µl of protease assay buffer (0.1 M sodium acetate, 1.0 M sodium chloride, 1.0 mM EDTA, 1.0 mM DTT, pH 4.7) containing 2.5 µM of either gag-pol or caspase 8 substrate peptide prewarmed to 37°C. Fluorescence intensity readings at 490 nm (excitation 340 nm) commenced immediately and data was continuously recorded every 2 minutes for 60 minutes.

### HIV-1 PR wild type or mutant protease expression

The PCR approaches were utilized to generate HIV-1 PR wild type or mutant sequences from PR expression vector pEYEP-C1 using forward primer 5′- AGAAGAGAGGATCCGATGTGGGGTAGA -3′ and reverse primer 5′- TTCTTC TGTGAATTCTCATTGTTTAAC - 3′. The PCR product was digested with BamH1 and EcoR1 (New England BioLabs, Ipswich, MA), and ligated into the protein expression vector pET-21b (EMD, Gibbstown, NJ) using T4 DNA ligase (Promega, Madison, WI), which had been restriction digested with BamH1 and EcoR1. The ligation product was transformed into E-coli stain BL21(DE3)pLysS (EMD, Gibbstown, NJ) cells. The insert was confirmed by DNA sequencing, using dye terminator cycle sequencing at Mayo DNA sequencing core facility. Proteases were expressed by an induction of 1.0 mM isopropyl beta-D thiogalactoside and bacterial pellet lysed directly using HIV-1 PR assay buffer, in order to avoid protease degradation with appropriate control cells lysate. The lysate were subjected to protease assay, as described.

### Statistical analysis

Values of continuous variables are expressed as means +/− standard error. Comparisons of continuous variables were made using T tests. The prevalence of resistance mutations in discordant versus concordant patients was compared using the Fisher's Exact test. A P value of ≤0.05 was considered statistically significant.
